# Gantry-free cone-beam CT arthrography for diagnosis of scapholunate ligament injuries: accelerating the preoperative work-up in acute wrist trauma

**DOI:** 10.1007/s00330-025-11405-7

**Published:** 2025-02-01

**Authors:** Karsten Sebastian Luetkens, Andreas Steven Kunz, Mila Marie Paul, Stefanie Hölscher-Doht, Henner Huflage, Julius Frederik Heidenreich, Lukas Müller, Thorsten Alexander Bley, Rainer Schmitt, Jan-Peter Grunz

**Affiliations:** 1 Department of Diagnostic and Interventional Radiology, University Hospital Würzburg, Oberdürrbacher Straße 6, 97080 Würzburg, Germany; 2 Department of Trauma, Hand, Plastic, and Reconstructive Surgery, University Hospital Würzburg, Oberdürrbacher Straße 6, 97080 Würzburg, Germany; 3 Department of Radiology, University of Wisconsin–Madison, 600 Highland Avenue, Madison, WI 53792 USA; 4 Department of Diagnostic and Interventional Radiology, University Hospital Mainz, Langenbeckstraße 1, 55131 Mainz, Germany; 5 Department of Radiology, University Hospital LMU Munich, Marchioninistraße 15, 81377 Munich, Germany

**Keywords:** Scapholunate ligament, Distal radius fracture, Cone-beam CT, Wrist arthrography, Diagnostic accuracy

## Abstract

**Objective:**

Combining fluoroscopy and high-resolution cone-beam CT (CBCT) in a multipurpose scanner without a conventional gantry holds the potential for time-saving in musculoskeletal interventions. This study investigated the performance of gantry-free CBCT arthrography in patients with suspected scapholunate ligament (SLL) tears.

**Materials and methods:**

Fifty-five patients (29 men, 46.0 ± 15.3 years) who underwent preoperative gantry-free CBCT arthrography between June 2021 and March 2024 were analyzed retrospectively. Three radiologists assessed CBCT arthrograms for tears of the palmar and dorsal SLL segments. Surgical reports served as the reference standard for calculating indicators of diagnostic accuracy. Interreader agreement was tested by computing Krippendorff *α*. Radiation dose and examination time were recorded.

**Results:**

Tears of the palmar and dorsal SLL segment were recorded in 25 (45%) and 6 patients (11%), respectively. CBCT arthrography facilitated good sensitivity (range for all readers: 84–92%) and excellent specificity (93–97%) in the assessment of the palmar SLL. For the dorsal SLL, sensitivity (83–100%) and specificity (96–98%) were even higher. Substantial agreement was determined for both the palmar (*α* = 0.83, 95% CI: 0.74–0.90) and dorsal SLL (0.84, 0.70–0.95). The mean volume CT dose index for CBCT arthrography was 3.2 ± 1.4 mGy. Not requiring patient repositioning, the median time between fluoroscopy-guided contrast injection and CBCT was 3:05 min (2:31–3:50 min).

**Conclusion:**

Gantry-free CBCT arthrography allows for excellent accuracy in the preoperative diagnosis of SLL tears with low radiation dose. The ability to alternate between fluoroscopy and CBCT without repositioning facilitates a “one-stop-shop” approach with short examination time.

**Key Points:**

***Question*** *Performing fluoroscopy-guided arthrography and high-resolution cone-beam CT without patient repositioning appears advantageous for the preoperative work-up of distal radius fractures with concomitant scapholunate ligament injuries*.

***Findings*** *Gantry-free cone-beam CT arthrography allowed for short examination times and high diagnostic accuracy for either segment of the scapholunate ligament (89–98% versus surgery)*.

***Clinical relevance*** *Preoperative assessment of scapholunate instability influences treatment since surgeons can reduce radius fractures and perform osteosynthesis via a dorsal portal to simultaneously stabilize the scapholunate compartment or use an additional dorsal access route for ligament suture and transfixation*.

## Introduction

Distal radius fractures are among the most common reasons for admission to emergency departments worldwide [1]. Depending on the fracture pattern and the degree of displacement, conventional radiography is often insufficient for surgical planning [2]. While cross-sectional imaging is valuable for 3D visualization of fracture morphology, conventional CT is incapable of displaying concomitant soft tissue injuries, for example, of the intrinsic carpal ligaments. Among these, tears of the scapholunate ligament (SLL) are most common, occurring in one of ten distal radius fractures, especially in the presence of radiocarpal joint surface affliction [3]. An intact SLL possesses a horseshoe-like appearance, connecting the scaphoid and lunate bone with a thick dorsal and a thinner palmar ligament segment [4, 5]. The dorsal SLL segment, in particular, is of relevance to carpal biomechanics, serving as the primary stabilizer of the proximal carpal row [6]. Insufficiency of this segment is mostly trauma-associated and frequently leads to dissociation in the scapholunate compartment, characterized by dorsal extension of the lunate (“dorsal intercalated segment instability”) and palmar rotatory subluxation of the scaphoid [7, 8].

Since non-surgical assessment of ligament integrity requires either MRI or arthrography in combination with cross-sectional imaging in the form of CT or MR arthrograms, SLL injuries are commonly overlooked in acute trauma settings [9, 10]. The reason is simple: Whereas unstable fracture patterns mandate timely reduction and fixation, SLL-specific imaging techniques are often not available in the preoperative workup of distal radius fractures. Due to their relevance for biomechanical function, however, untreated SLL tears may induce a loss of carpal height and secondary osteoarthritis over time, a condition known as “scapholunate advanced collapse” [11]. To prevent insufficient preoperative assessment of SLL integrity in the acute trauma setting, a time-efficient yet accurate diagnostic instrument is warranted.

With the expansion of cone-beam CT (CBCT) from maxillofacial to musculoskeletal imaging in the last decade [12–15], such an instrument may now be available in the form of CBCT arthrography. Combining fluoroscopy and high-resolution 3D imaging in one system, the introduction of dedicated extremity scanners without a conventional gantry has opened up new options for the workup of distal radius fractures [16, 17]. Despite its theoretical advantages over conventional CT and MR arthrograms regarding spatial resolution and diagnostic workflow, in-vivo data on the actual clinical value of CBCT arthrography is still lacking.

This study was designed to investigate the diagnostic accuracy and time-saving potential of gantry-free CBCT arthrography in patients with suspected SLL tears after acute wrist trauma.

## Materials and methods

### Patients

Approval for this retrospective single-center investigation was obtained from the local institutional review board, which waived the need for additional written informed consent (20210615 01). Between June 2021 and July 2024, 94 consecutive patients were referred to the radiology department of a tertiary-care university hospital for CBCT arthrography with a multipurpose, gantry-free x-ray system (Multitom Rax, Siemens Healthineers). Exclusion criteria for this study were defined as follows: no acute trauma history, extensive motion artifacts, absence of contrast agent in the radiocarpal compartment, and no surgical assessment of SLL integrity. For each included individual, sex, age (years), laterality, and time to imaging (days between trauma and CBCT arthrography) were noted. In addition, the presence or absence of a distal radius fracture was recorded as well as information on multi-fragmentary fracture patterns and/or radiocarpal surface affliction.

### Direct arthrography

Arthrograms were performed by board-certified radiologists with seven to ten years of training in the field using the fluoroscopy mode of the twin robotic x-ray system to localize the optimal injection sites. Fluoroscopy guidance employed a median tube voltage of 65 kV and was regulated with the system’s multifunctional footswitch. Depending on their mobility and pain level, patients adopted either a prone or supine position with the arm abducted by approximately 90° and the hand in pronation. In order to assess the integrity of the intrinsic carpal ligaments, the midcarpal articulation was contrasted first with the injection site being located dorsally between the lunate, triquetral, capitate, and hamate bone. Afterward, the radiocarpal joint was punctured close to the proximal scaphoid pole. A half-half mixture of iodine contrast medium with a concentration of 300 mg per mL (Imeron 300, Bracco) and anesthetic with a concentration of 10 mg per mL (Mecain, Puren Pharma) was injected. Injected volumes for the midcarpal and radiocarpal joint each ranged between 2 and 4 mL.

### Cone-beam CT

For subsequent CBCT imaging, patients remained in the position they had adopted for the fluoroscopy-guided injection of contrast agent. This “one-stop-shop” approach exploits the multifunctional capabilities of the twin robotic system, which are realized by an open scanner architecture instead of a conventional CT gantry. The setup includes two telescopic arms mounted on ceiling rails, which carry the x-ray tube and flat-panel detector, respectively. Using a trajectory of synchronized arm movement, wrists were scanned with 80.3 kV, an asymmetrical source-to-image distance of 115 cm, and a sweep angle of 200°. Un-binned readout of the system’s flat-panel detector in high-resolution mode results in a 1440 × 1440 pixel matrix. The acquired projection images were reconstructed using a scanner-side workstation with specific software (syngo.via View&GO, Siemens Healthineers). Post-processing employed a dedicated high-resolution bone kernel with a modulation transfer function comparable to state-of-the-art multidetector CT (*ρ*_50_ = 16.7 line pairs per cm, *ρ*_10_ = 25.4 line pairs per cm) in a 100 mm field of view. In addition to axial thin slices of 0.5 mm for manual angulated multiplanar reformatting, three-planar reconstruction of data was performed with a slice thickness and increment of 1 mm, respectively. Window levels were preset to 3000 and 1000 Hounsfield units (width and center). Dose-are products for fluoroscopy and CBCT as well as volume CT dose indices (calculated for a 16 cm dosimetry phantom) were obtained from the automatically-generated scanner report, while the interval between contrast injection and the completion of CBCT imaging was recorded based on time stamps within the DICOM data.

### Diagnostic assessment

Three board-certified radiologists with at least 7 years of experience in musculoskeletal imaging (R1, K.S.L.; R2, A.S.K.; R3, J.-P.G.) read all CBCT arthrograms independently and in randomized order using the clinical PACS system (Merlin, Phönix-PACS). Blinded to any patient-specific information, observers were tasked to separately analyze the structural integrity of the palmar and dorsal SLL segment in a dichotomous fashion (i.e., tear/no tear). Intraoperative SLL assessment by direct visual inspection as well as by dynamic fluoroscopy after plate osteosynthesis of the distal radius served as the reference standard for ligament integrity and for the calculation of diagnostic accuracy in all patients.

### Statistical analysis

J.-P.G. performed data analyses using dedicated statistical software (SPSS Statistics Version 29.0.1, IBM). The normal distribution of continuous items was controlled with the Shapiro-Wilk test. Nonparametric variables are reported as absolute and relative frequencies with median and interquartile range values, whereas normally distributed data are presented as mean ± standard deviation. Calculation and presentation of diagnostic performance indicators (i.e., specificity, sensitivity, and accuracy) follow the Standards for Reporting of Diagnostic Accuracy (STARD). Interreader reliability was ascertained by computing Krippendorff *α* with 95% confidence intervals (95% CI). Interpretation followed Hayes and Krippendorff with *α* ≥ 0.80 being deemed representative of good interreader reliability [18].

## Results

### Patient characteristics

**Fig. 1:**
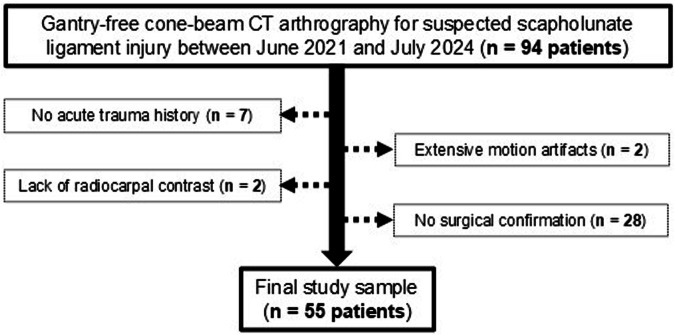
Flow chart highlighting the study inclusion and exclusion criteria

Exclusion of 39 patients (Fig. 1; no acute trauma history, *n* = 7; extensive motion artifacts, *n* = 2; absence of contrast agent in the radiocarpal compartment, *n* = 2; no surgical confirmation of SLL integrity, *n* = 28) resulted in a final study sample of 55 individuals (mean age, 46.0 ± 15.3 (SD) years, 26 women, 29 men). The median time interval between trauma and imaging was 3 days (interquartile range 1–4.5 days). Extension trauma was recorded in 35 patients, while flexion and direct trauma were noted in 10 patients each (Table 1). Injuries of the palmar and dorsal SLL were surgically determined in 25 and 6 patients, respectively. Applying a modified version of the EWAS criteria for SLL injuries [19], stage III A was most common among injured wrists (Supplementary Table S1). Complete articular radius fractures (AO type C, 35 patients) were substantially more common than partial articular (AO type B, 17 patients) and extraarticular fractures (AO type A, 3 patients) (Table 2).

**Table 1:** Patient sample

Study sample	55 patients
Sex	
Women	26 (47%)
Men	29 (53%)
Age	
Mean ± standard deviation	46.0 ± 15.3 years
Range	17–66 years
Laterality	
Left wrist	27 (49%)
Right wrist	28 (51%)
Acute trauma	
Time to CBCT arthrography	3 [1–4.5] days
Extension trauma	35 (64%)
Flexion trauma	10 (18%)
Direct trauma	10 (18%)
Scapholunate ligament tears	
SLL tear (any)	26 (47%)
Palmar SLL segment tear	25 (45%)
Dorsal SLL segment tear	6 (11%)

Continuous data are provided as mean ± SD, while the absolute number of patients is given for dichotomous items with percentages in parentheses. For nonparametric data, the median value is provided with the interquartile range in brackets. *CBCT* cone-beam CT, *SLL* scapholunate ligament

**Table 2:** AO classification of distal radius fractures

Radius fracture (any)	55 (100%)
AO type A (extraarticular) fracture	3 (5%)
A1 (radial styloid avulsion)	0 (0%)
A2 (simple)	2 (4%)
A3 (wedge or multi-fragment)	1 (2%)
AO type B (partial articular) fracture	17 (31%)
B1 (sagittal)	10 (18%)
B2 (dorsal rim)	2 (4%)
B3 (volar rim)	5 (9%)
AO type C (complete articular) fracture	35 (64%)
C1 (simple articular and metaphyseal)	10 (18%)
C2 (metaphyseal multi-fragment)	11 (20%)
C3 (articular multi-fragment and metaphyseal)	14 (25%)

The absolute number of fractures is reported with percentages in parentheses

### Diagnostic assessment of the palmar scapholunate ligament

**Fig. 2:**
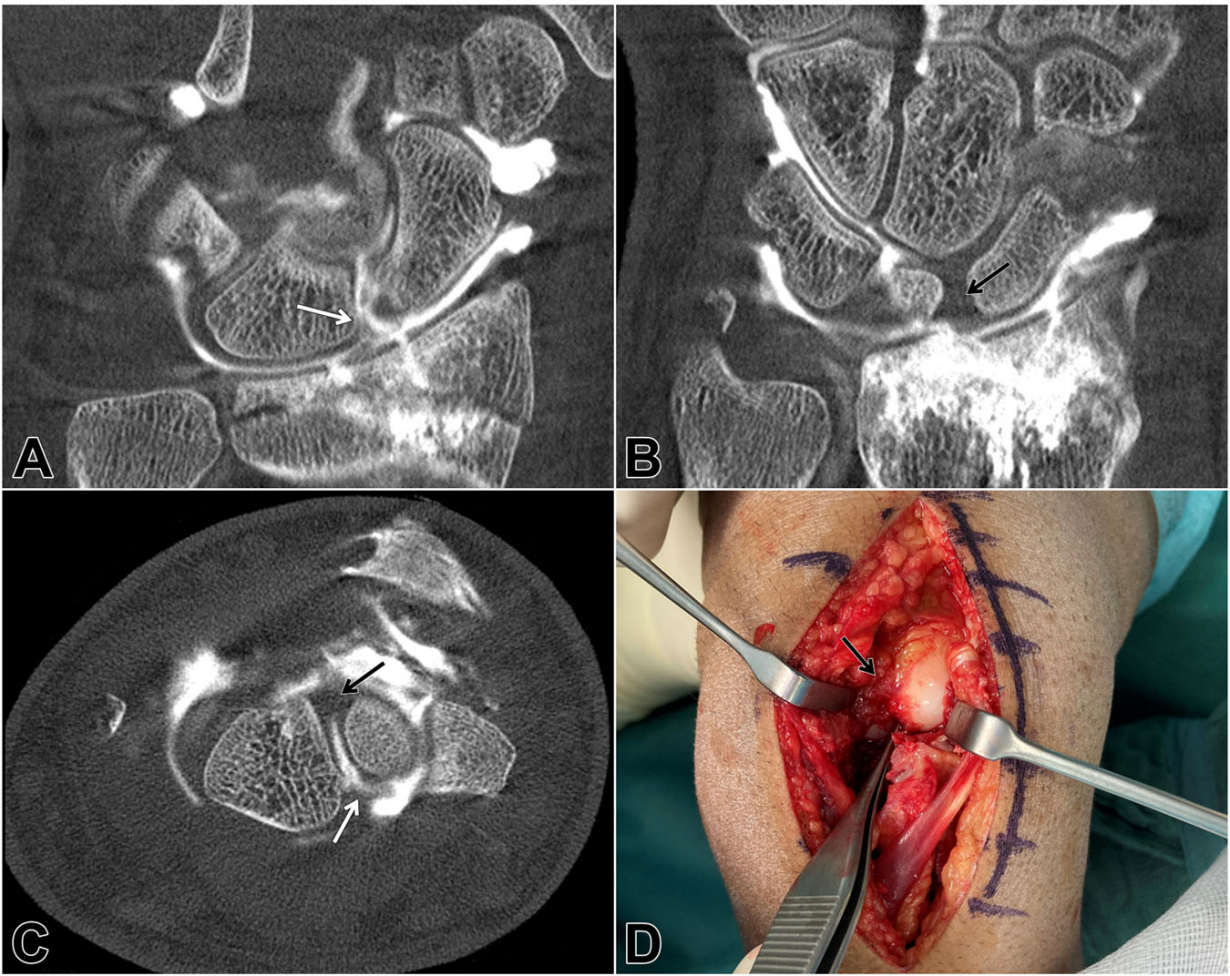
Cone-beam CT arthrography was requested for a 29-year-old man with an articular, multi-fragmentary radius fracture after a fall from a great height. CT images after articular contrast injection revealed a tear of the palmar scapholunate ligament (**A**: coronal, **C**: axial; *white arrows*), whereas the stabilizing dorsal segment was deemed intact (**B**: coronal, **C**: axial; *black arrows*). Undergoing open reduction and internal fixation of the radius fracture, no additional ligament suture or carpal transfixation was performed based on the integrity of the dorsal scapholunate ligament (**D**; *black arrow*)

Classification functions of diagnostic test accuracy are summarized in Table 3. Gantry-free CBCT arthrography allowed for excellent specificity (R1: 97% [95% CI: 83–100%]; R2: 97% [95% CI: 83–100%]; R3: 93% [95% CI: 78–99%]) and good sensitivity in the assessment of the palmar SLL (R1: 92% [95% CI: 74–99%]; R2: 88% [95% CI: 69–97%]; R3: 84% [95% CI: 64–95%]). Interreader reliability was high (Krippendorff *α* = 0.83, 95% CI: 0.74–0.90). An example of a distal radius fracture with concomitant palmar SLL injury is given in Fig. 2.

**Table 3:** Scapholunate ligament tear analysis

	Reader 1	Reader 2	Reader 3
SLL (any)			
Specificity	93 [77–99] (27/29)	97 [82–100] (28/29)	97 [82–100] (28/29)
Sensitivity	92 [75–99] (24/26)	88 [70–98] (23/26)	85 [65–96] (22/26)
Accuracy	93 [82–98] (51/55)	93 [82–98] (51/55)	91 [80–97] (50/55)
Palmar SLL segment			
Specificity	97 [83–100] (29/30)	97 [83–100] (29/30)	93 [78–99] (28/30)
Sensitivity	92 [74–99] (23/25)	88 [69–97] (22/25)	84 [64–95] (21/25)
Accuracy	95 [85–99] (52/55)	93 [82–98] (51/55)	89 [78–96] (49/55)
Dorsal SLL segment			
Specificity	98 [89–100] (48/49)	96 [86–100] (47/49)	98 [89–100] (48/49)
Sensitivity	100 [54–100] (6/6)	83 [36–100] (5/6)	100 [54–100] (6/6)
Accuracy	98 [90–100] (54/55)	95 [85–99] (52/55)	98 [90–100] (54/55)

Indicators of diagnostic performance are reported with 95% confidence intervals in brackets and absolute frequencies in parentheses. *SLL* scapholunate ligament

### Diagnostic assessment of the dorsal scapholunate ligament

**Fig. 3:**
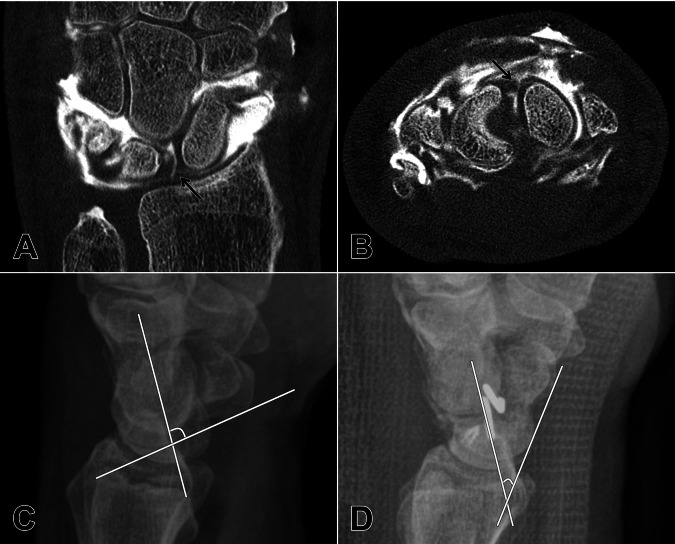
Suffering a motorcycle accident, a 62-year-old man was admitted to the emergency department for increasing left wrist pain and swelling. Cone-beam CT arthrography revealed a destabilizing tear of the scapholunate ligament’s dorsal segment (**A**/**B**, *black arrows*), while the previously taken radiograms displayed a widened scapholunate angle indicative of dorsal intercalated segment instability (**C**). Without patient repositioning between contrast injection into the radiocarpal compartment under fluoroscopic guidance and the completion of 3D imaging, the entire procedure required less than 5 min time. Subsequently, the patient underwent ligament suture as well as scapholunate and scaphocapitate transfixation to re-establish the stability of the proximal carpal row. Surgical success is indicated by marked narrowing of the scapholunate angle in the postoperative lateral radiogram (**D**)

**Fig. 4:**
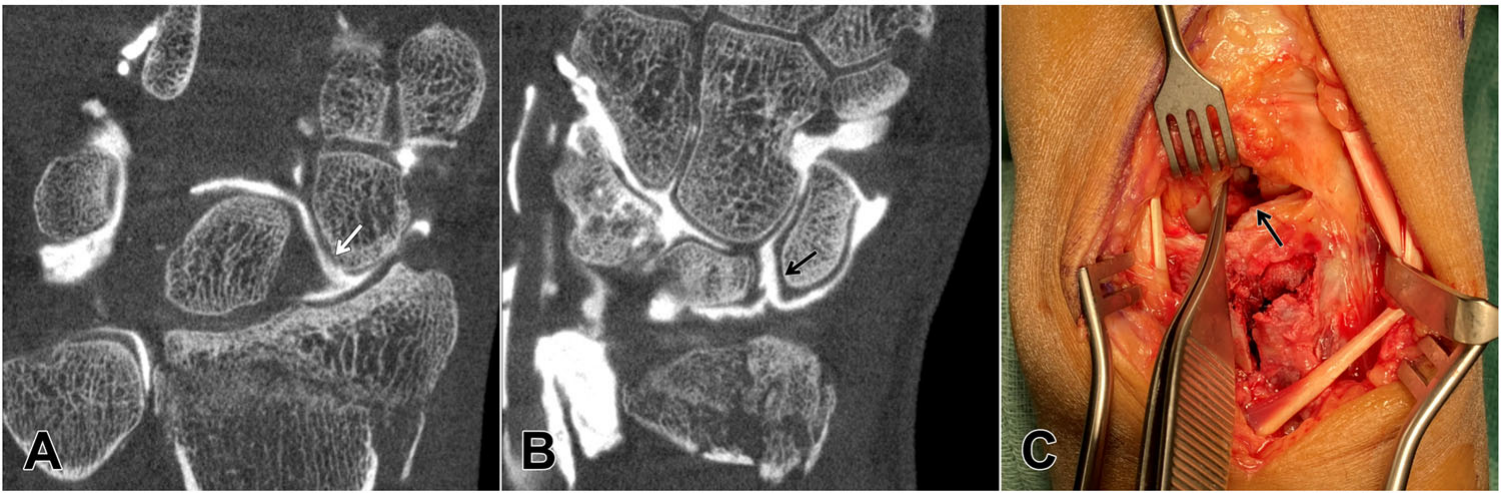
During ice skating, a 54-year-old goldsmith fell on his outstretched arm and suffered a distal radius fracture with a multi-fragmentary pattern that involved both the radiocarpal and distal radioulnar joint surface. Due to the bi-articular fracture morphology, cone-beam CT arthrography was requested preoperatively to assess the ligamentous integrity of the proximal carpal row. The minimally invasive examination displayed both the complicated fracture pattern as well as tears of the palmar (**A**; *white arrow*) and dorsal scapholunate ligament (**B**; *black arrow*). Preoperative knowledge of the destabilizing ligament rupture resulted in a dorsal approach for fracture reduction and plate fixation that allowed for simultaneous refixation of the torn scapholunate ligament (**C**; *black arrow*)

For the dorsal SLL, the diagnostic specificity of gantry-free CBCT arthrography was even higher than for the palmar segment (R1: 98% [95% CI: 89–100%]; R2: 96% [95% CI: 86–100%]; R3: 98% [95% CI: 89–100%]). One reader missed a single dorsal SLL rupture, resulting in almost perfect sensitivity (R1: 100% [95% CI: 54–100%]; R2: 83% [95% CI: 36–100%]; R3: 100% [95% CI: 54–100%]). Figure 3 shows an isolated tear of the dorsal SLL, whereas Fig. 4 highlights a complete tear involving all ligament segments. Interreader reliability was slightly better than for assessment of the palmar ligament segment (Krippendorff *α* = 0.84, 95% CI: 0.70–0.95).

### Radiation dose and examination time

The dose-area products for fluoroscopy and CBCT were 12.1 ± 22.6 µGy × m^2^ and 112.9 ± 51.1 µGy × m^2^, respectively, while the volume CT dose index for the latter was 3.1 ± 1.4 mGy. Without patient repositioning in between, the median time interval between fluoroscopy for the guidance of the articular contrast injection and the completion of CBCT imaging was 3:05 min (interquartile range 2:31–3:50 min).

## Discussion

In this retrospective investigation of 55 individuals with acute wrist trauma, we studied the performance of high-resolution gantry-free cone-beam CT arthrography in the preoperative diagnosis of SLL injuries. Without patient repositioning between the fluoroscopy-guided multicompartment injection of contrast agent and subsequent 3D imaging of the wrist, cone-beam CT arthrography allowed for time-efficient “one-stop-shop” imaging of bone and ligament injuries with very low radiation dose. Using surgical confirmation as the standard of reference in all subjects, our results suggest that preoperative cone-beam CT arthrography is highly accurate in detecting and ruling out SLL injuries, particularly of its dorsal segment, which serves as the primary stabilizer of the proximal carpal row.

While studies have previously shown the benefits of direct arthrography in combination with cross-sectional imaging for intrinsic carpal ligament injuries [3, 20, 21], the use of a conventional gantry-based scanner entails major disadvantages for trauma patients that can be overcome with the multifunctional setup investigated in the current study: First, gantry-free CBCT arthrography is performed in a comfortable tableside scan position that can easily be adopted, even in the presence of disabilities or concomitant injuries of the arm and shoulder [22]. This is in contrast to the so-called ‘superman position’ required for optimal image quality and dose reduction in multidetector CT, which may be difficult to obtain for patients in pain [23]. Second, the twin robotic system facilitates both fluoroscopy and CBCT in the same imaging session. Whereas multidetector CT or MR arthrography requires not only repositioning but also patient transfer from the fluoroscopy suite to the respective scanner room, the open architecture of the employed multipurpose system allows individuals to maintain their stance for the entire imaging procedure. Thereby, scheduling conflicts between scanners can be avoided and examination time can be substantially reduced, which limits both resorption and extraarticular diffusion of contrast agent [16]. Especially in comparison with MR arthrography, the reduction in examination time with CBCT arthrography is considerable [24, 25], potentially influencing the clinical workflow by freeing additional MRI capacities for other imaging tasks.

In recent literature, multidetector CT arthrography has been reported to be as accurate as MR arthrography for diagnosing cartilage lesions, intrinsic carpal ligament tears, and ulnocarpal complex injuries [20, 21, 26]. In contrast to its advantages in the detection of bone marrow abnormalities and synovitis, MRI has been shown to be of limited value in the detection of subtle chondral lesions and partial ligament tears due to its lower spatial resolution compared with CT [9]. By combining an asymmetric acquisition geometry to counteract the influence of focal spot size with un-binned flat-panel detector readout, gantry-free CBCT arthrography realizes an isotropic voxel size of 0.15 mm [22]. This facilitates angulated multiplanar reconstructions that account for the oblique course of the SLL, subsequently offsetting the majority of partial volume effects. Our findings suggest that the resulting images allow for excellent diagnostic performance in the assessment of the SLL.

Preoperative knowledge of SLL instability directly influences treatment since surgeons can perform radius fracture reduction and internal fixation primarily via a dorsal access route to stabilize the scapholunate compartment through the same portal [27] or use an additional dorsal portal for SLL suture and transfixation [28]. Although wrist arthroscopy can be added to open surgical procedures [29, 30], literature does not consistently suggest that arthroscopic assistance improves the reduction outcome [31]. Unlike the cases in this investigation, not every SLL rupture occurs in combination with a distal radius fracture, though. In patients where no open surgery is required for fracture treatment, wrist arthroscopy constitutes the established standard for operative SLL assessment and reconstruction [32]. However, arthroscopy should primarily be reserved for suspected SLL injuries with biomechanical relevance, i.e., ruptures that involve the dorsal ligament portion [33]. Whether high-resolution CBCT arthrography is similarly effective in patients without concomitant radius fractures should be the focus of future studies.

Notably, despite the acquisition time of 14 s per 3D scan, the required radiation dose for diagnostic image quality with gantry-free CBCT arthrography was markedly low at 3.1 ± 1.4 mGy. Although the appendicular skeleton is considered less radiation-sensitive than the body trunk [34], the ALARA principle commands radiologists to maintain the lowest reasonably achievable dose for any given imaging task. In line with prior investigations on multidetector CT versus CBCT arthrography, the radiation exposure in the present study was substantially lower than with a conventional CT scanner [25]. Supporting the findings of Saupe et al [35], no joint infections or other severe side effects were recorded after the minimally invasive examinations. It may be noted that the relatively long scan time holds potential for motion artifacts, however, the small number of artifact-related study exclusions (*n* = 2 of 94, 2.1%) underlines the effectiveness of comfortable tableside positioning and motion compensation algorithms.

Several methodological limitations must be recognized when interpreting the study’s results. Patients did not receive a second form of cross-sectional imaging after multicompartment arthrography, therefore no intraindividual comparison to multidetector CT or MR arthrograms was possible. Although not measured for the investigated patient sample, the median time required for the automated scanner-side image reconstruction and PACS transfer was 2.4 min in an earlier cadaveric feasibility study [16]. Since a surgical reference standard was considered mandatory for study inclusion, a certain selection bias towards more severe wrist injuries with displaced fracture patterns was inevitable. While the resulting absence of uninjured wrists and the high pretest probability for SLL lesions within the study sample are acknowledged as limitations, we believe that the performance of CBCT arthrography should be held to the highest possible standard of reference in the form of intraoperative assessment.

In conclusion, gantry-free cone-beam CT arthrography allows for excellent diagnostic accuracy in the detection and exclusion of SLL tears with low radiation dose. The ability to alternate between fluoroscopy and cone-beam CT without patient repositioning facilitates a “one-stop-shop” approach with potential for considerable examination time saving.

## Disclaimer

For regulatory reasons, Multitom Rax and the software version VF11 are not commercially available in all countries, and their future availability cannot be guaranteed.

## Supplementary information

ELECTRONIC SUPPLEMENTARY MATERIAL

## Abbreviations

CBCT: Cone-beam computed tomography
SLL: Scapholunate ligament
