# Supplementary material for: Gantry-free cone-beam CT arthrography for diagnosis of scapholunate ligament injuries: accelerating the preoperative work-up in acute wrist trauma
Source: Eur Radiol. 2025 Feb 1;35(8):4562–70. doi: 10.1007/s00330-025-11405-7 (PMC12226655; doi:10.1007/s00330-025-11405-7)
Supplement: Supplementary file 1 — ELECTRONIC SUPPLEMENTARY MATERIAL [file 330_2025_11405_MOESM1_ESM.pdf]

Gantry-free cone-beam CT arthrography for diagnosis of scapholunate ligament injuries: Accelerating the preoperative work-up in acute wrist trauma

ELECTRONIC SUPPLEMENTARY MATERIAL

Supplementary Table 1 – Classification of scapholunate ligament injuries according to the modified EWAS criteria

Stage	Explanation	n (%)
**I**	No SLL lesion	21 (38%)
**II**	Only membranous pinhole lesion of the SLL	8 (15%)
**III A**	Partial lesion involving the volar SLL	20 (36%)
**III B**	Partial lesion involving the dorsal SLL	1 (2%)
**III C**	Complete SLL tear, dynamic injury	2 (4%)
**IV**	Complete SLL tear, static injury with small joint gap	3 (5%)
**V**	Complete SLL tear, static injury with wide joint gap	0 (0%)

Note. – *Since the EWAS system is based on arthroscopic findings, its criteria were modified to account for the open surgical approach used to treat the patients included in this study. The absolute number of lesions is reported with percentages in parentheses.* ***EWAS*** *= European Wrist Arthroscopy Society;* ***SLL*** *= scapholunate ligament.*
